# Transversus abdominis release for complex incisional hernias—a case report

**DOI:** 10.1093/jscr/rjab281

**Published:** 2021-07-14

**Authors:** Sunil Basukala, Rakesh Kumar Gupta, Narayan Thapa, Bikash Bahadur Rayamajhi, Raveesh Mishra, Pankaj Mandal

**Affiliations:** Department of Surgery, Nepal Army Institute of Health Science (NAIHS), Kathmandu, Nepal; Department of Surgery, BP Koirala Institute of Health Sciences (BPKIHS), Dharan, Nepal; Department of Surgery, Nepal Army Institute of Health Science (NAIHS), Kathmandu, Nepal; Department of Surgery, Nepal Army Institute of Health Science (NAIHS), Kathmandu, Nepal; Department of Anaesthesiology, Nepal Army Institute of Health Science (NAIHS), Kathmandu, Nepal; Department of Surgery, Nepal Army Institute of Health Science (NAIHS), Kathmandu, Nepal

## Abstract

Complex ventral hernia repair has been a challenging task of difficulty in primary closure of the defects. Transversus abdominis muscle release (TAR) procedure, as a type of posterior component separation, is a new myofascial release technique in complex ventral hernia repair. TAR creates immense retro muscular plane and allows bilaminar ingrowth of the mesh, allowing primary closure of defect. Owing to its favorable outcome, suitability of TAR technique in treatment of complex ventral hernia could be explored further where closure of the primary defect is difficult.

## INTRODUCTION

The ideal surgical approach to the difficult ventral hernia repair is still a matter of debate because of the high perioperative morbidity (abdominal compartment syndrome and respiratory failure), frequent recurrences and poor quality of life. Recently described by European Hernia Association as a clear entity, difficult complex abdominal wall is a large challenge for both surgeon and patient. Closing such defects is a significant problem in obtaining a reliable, durable repair with low morbidity and recurrence rate [1, 2]. The concept of component separation technique (CST) for the treatment of very large primary and incisional abdominal wall hernias was developed because the traditional suture and mesh techniques without relaxing the musculofascial flaps lead to unfavorable results [3].

The classic open anterior CST was first published by Albanese and, decades later, popularized by Ramirez. The open anterior CST was developed for the reconstruction of a functional abdominal wall with autologous tissue repair [4]. As the number of large and complex abdominal wall defects is increasing, it is obvious that the procedure is not adequate for such pathology. Some modifications of the technique were reported, but the limited advancement of the rectus abdominis muscle makes them inappropriate. In 2012, Novitsky *et al*. [5] reported a novel approach to posterior component separation by transversus abdominis muscle release (TAR). This is a lateral extension of Rives–Stoppa repair with the creation of a wide space between the transversus abdominis muscle and fascia transversalis peritoneum complex [5, 6].

The goal of the paper is to present the operative technical details of the procedure and our short-term results.

## CASE REPORT

**Figure 1:**
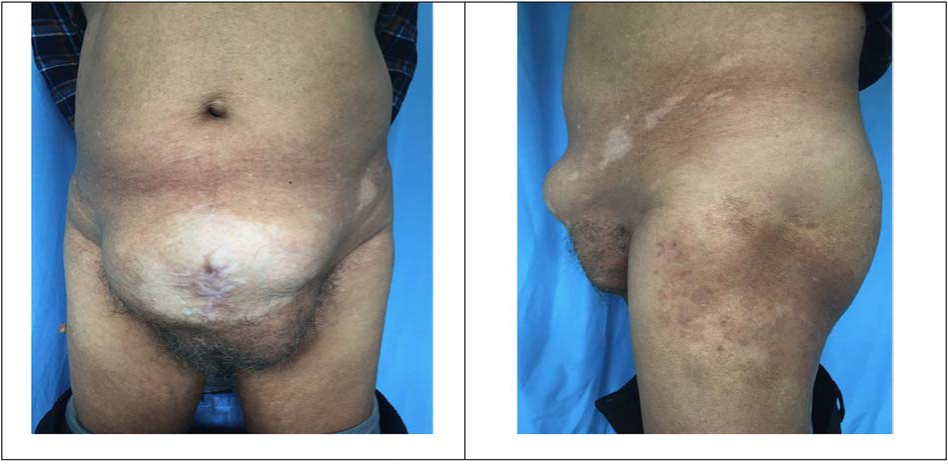
Protuberant lower midline incisional hernia before surgery.

A 68-year-old male, ex-serviceman, presented with a history of open prostatectomy (Millins). The procedure was operated elsewhere and presented with a complaint of a bulge in the abdomen along a previous scar site. He did not have any complaints of altered bowel and bladder habits. He did not have any other specific complaints. On evaluation, he gave history of surgery 1 year back. One year ago, he underwent Millins procedure for benign prostatic enlargement (BPE). This procedure involves removing part of the prostate using a transcapsular retro pubic approach (extra peritoneal) through a cut in the abdomen. Two weeks after the surgery, he developed abdominal wound dehiscence. Once the wound granulated well, he underwent secondary suturing of the abdominal wound after 2 months of surgery. He developed incisional hernia 3 months later. He continued to have protuberant abdomen with visible bowel peristalsis. He was reassured and advised to wear abdominal binder. On examination, his general condition was good. There was a bulge of size ~25 × 10 cm projecting from his anterior abdominal wall at the site of the previous scar (Fig. 1).

**Figure 2:**
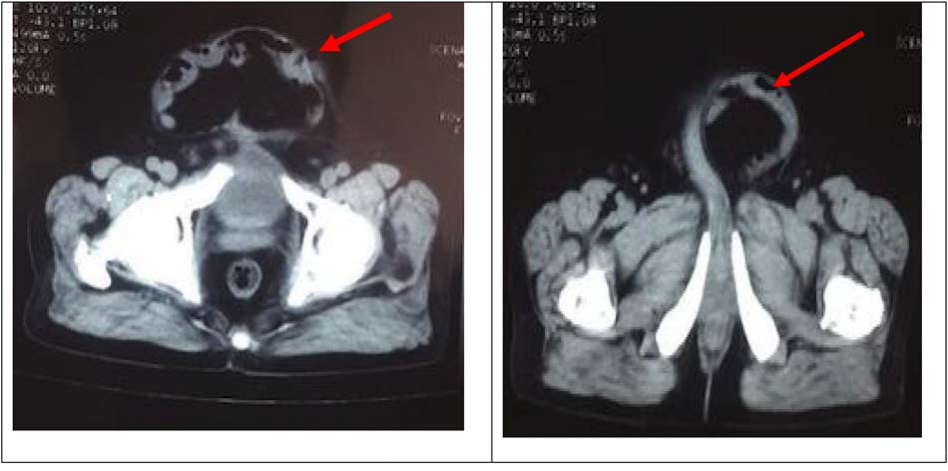
CT of the abdomen confirmed thinning of the rectus sheath with focal outpouching of rectus in the infra umbilical region and herniation of small bowel loops. Red arrows showing herniation of small bowel in infraumbilical region.

A palpable midline rectus defect of 10 cm was noted. Visible bowel peristalsis was seen. Other system examinations were normal. Routine laboratory investigations were normal, and he did not have any comorbid illness. Computed tomography (CT) of the abdomen confirmed thinning of the rectus sheath with focal outpouching of rectus in the infra-umbilical region and herniation of small bowel loops with loss of domain (LOD) (Fig. 2).

**Figure 3:**
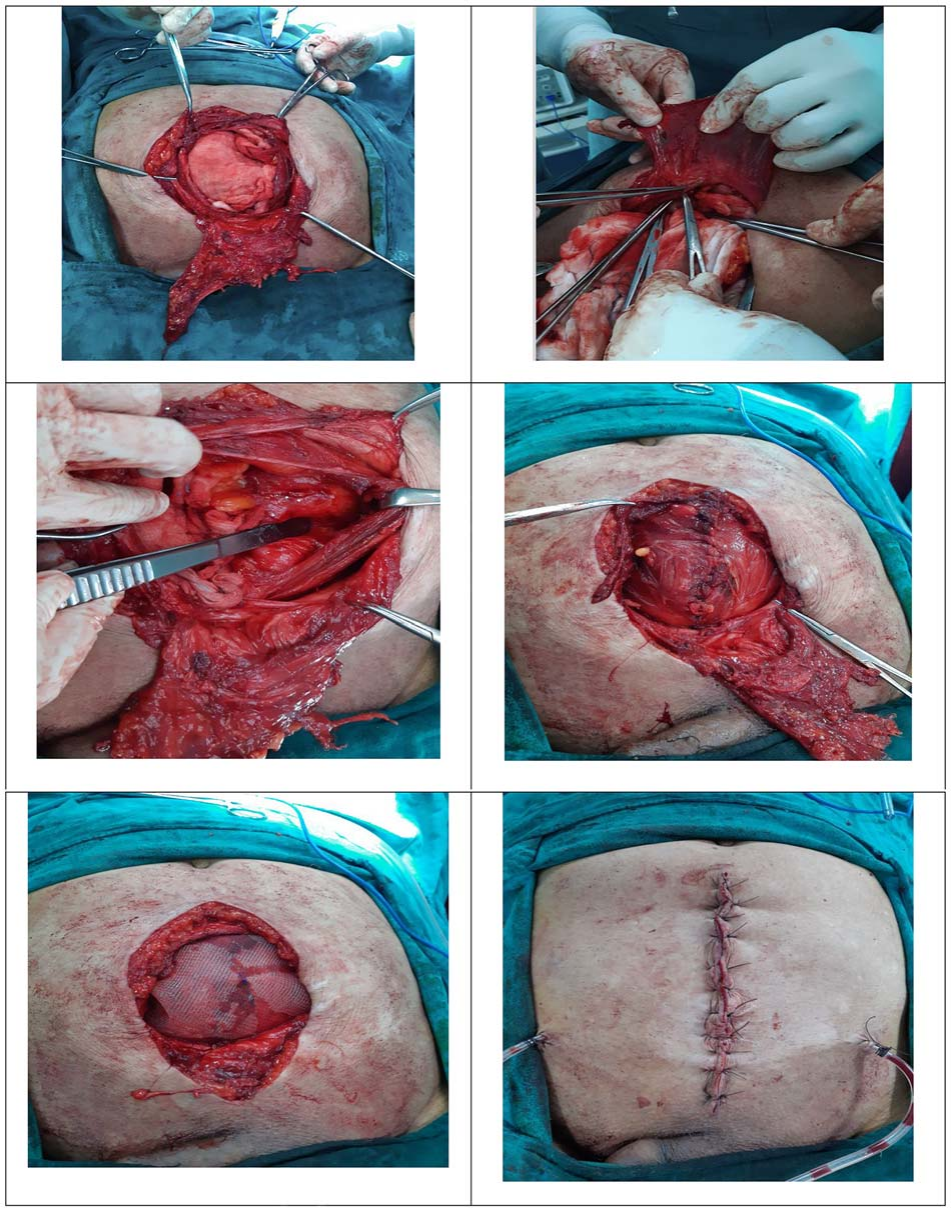
Incisional hernia with large sac; excision posterior rectus sheath; TAR; line of insertion of transversus abdominis; abdominal wound after skin approximation.

He was planned for surgical exploration. On exploration, a defect in midline for ~8 cm in width was noted. Rectus was retracted laterally and could not be brought easily to the midline. Adhesiolysis was done. It was decided to go ahead with posterior component separation with TAR since the defect was very wide. The procedure commenced with the separation of posterior rectus sheath from the anterior rectus at ~1 cm from the midline where the previous linea alba was present. Retro rectus dissection was done till the level of linea semilunaris. Care was taken to preserve the neurovascular bundles encountered. Incision was made on internal oblique fascia and the transverse abdominis muscle was hooked and divided using an electrocautery. The transverse abdominis muscle fibers were released along its entire insertion line at the level of semilunaris extending from xiphoid process above. Inferiorly, it was separated till the level of arcuate semilunaris below which the muscles were deficient and only peritoneum was present. Laterally, the release process was extended till bilateral psoas muscles were visualized. Superiorly, it was extended till the central tendon of diaphragm. The posterior rectus sheath was approximated in midline using non-absorbable sutures after placement of intra-peritoneal drains. Polypropylene mesh of size ~30 × 15 cm was placed over the posterior rectus sheath covering in a sublay fashion and was secured. Suction drain tubes were placed over the mesh covering and the anterior rectus sheath was approximated in the midline without tension. Skin closed in the midline (Fig. 3). Daily vitals and drain output was monitored. After considerable decrease in drain output, it was removed on the fourth post-operative day. Abdominal sutures were removed at the end of second post-operative week. He was subsequently discharged a week later and was put on abdominal binder. Patient attended his routine outpatient visit after 2 weeks with no complaints.

## DISCUSSSION

The classic Rives–Stoppa hernia repair, described way back in the 1970s, uses the potential space between the posterior rectus fascia and the rectus muscle. This technique has been time-tested and has proven to be an effective approach for open ventral hernia repairs. This allows placement of prosthetic mesh that extends ~6–8 cm on either side of the midline. However, there are certain circumstances where the defect is greater and Stoppa’s repair alone is not sufficient to achieve a tension-free midline approximation [6].

Novitsky, in 2012, introduced this new reconstructive technique of TAR and showed durable results with this technique. It is a modification of the Rives-Stoppa technique, which involves the retromuscular placement of mesh anterior to the posterior fascia and the primary closure of the anterior fascia. The extended retro rectus plane bounded by the psoas muscle laterally, central tendon of the diaphragm under the costal margin superolaterally, the inguinal ligament inferolaterally and space of Retzius inferiorly. This plane is utilized for mesh reinforcement and also to achieve a tension-free midline rectus closure [7].

TAR, as a type of posterior component separation, is a new myofascial release technique in complex ventral hernia repair. This modification allows for significant posterior rectus fascia advancement, wide lateral dissection, preservation of neurovascular supply, avoids subcutaneous tissue undermining and provides a large space for mesh sublay. TAR created an immense retromuscular plane and allows bilaminar ingrowth of the mesh. This novel technique for posterior component separation is associated with low perioperative morbidity and a low recurrence rate. Overall TAR may be an important addition to the armamentarium of surgeons undertaking major abdominal wall reconstructions [8–10].

## CONCLUSION

TAR is a novel technique of abdominal wall reconstruction which is a modification of a posterior component separation for repair of complex incisional hernia. TAR is associated with low perioperative morbidity, wound related complications and a low recurrence rate. TAR appears to be a safe and effective method for complex incisional hernia repair, which should be a valuable addition to the armamentarium of surgeons performing complex abdominal wall reconstructions.
